# It’s in the eye of the beholder: selective attention to drink properties during tasting influences brain activation in gustatory and reward regions

**DOI:** 10.1007/s11682-017-9710-2

**Published:** 2017-03-20

**Authors:** Inge van Rijn, Cees de Graaf, Paul A. M. Smeets

**Affiliations:** 1 Division of Human Nutrition, Wageningen University & Research, Stippeneng 4, 6708 WE Wageningen, The Netherlands; 2 Image Sciences Institute, University Medical Center Utrecht, Heidelberglaan 100, 3584 CX Utrecht, The Netherlands

**Keywords:** Selective attention, Functional magnetic resonance imaging, Taste, Intensity, Pleasantness, Calories

## Abstract

Statements regarding pleasantness, taste intensity or caloric content on a food label may influence the attention consumers pay to such characteristics during consumption. There is little research on the effects of selective attention on taste perception and associated brain activation in regular drinks. The aim of this study was to investigate the effect of selective attention on hedonics, intensity and caloric content on brain responses during tasting drinks. Using functional MRI brain responses of 27 women were measured while they paid attention to the intensity, pleasantness or caloric content of fruit juice, tomato juice and water. Brain activation during tasting largely overlapped between the three selective attention conditions and was found in the rolandic operculum, insula and overlying frontal operculum, striatum, amygdala, thalamus, anterior cingulate cortex and middle orbitofrontal cortex (OFC). Brain activation was higher during selective attention to taste intensity compared to calories in the right middle OFC and during selective attention to pleasantness compared to intensity in the right putamen, right ACC and bilateral middle insula. Intensity ratings correlated with brain activation during selective attention to taste intensity in the anterior insula and lateral OFC. Our data suggest that not only the anterior insula but also the middle and lateral OFC are involved in evaluating taste intensity. Furthermore, selective attention to pleasantness engaged regions associated with food reward. Overall, our results indicate that selective attention to food properties can alter the activation of gustatory and reward regions. This may underlie effects of food labels on the consumption experience of consumers.

**Electronic supplementary material:**

The online version of this article (doi:10.1007/s11682-017-9710-2) contains supplementary material, which is available to authorized users.

## Introduction

Selective attention to one specific food property over another may alter the taste perception of a food (Liem et al. 2012a, 2012b). In daily life, attention of consumers is often directed towards a specific property by product labels that emphasize either the hedonics, sensory characteristics or caloric content (Borgmeier and Westenhoefer 2009). Such product labels may, in turn, influence consumers’ buying and eating behavior (Bushman 1998; Miller et al. 1998; Westcombe and Wardle 1997). Better understanding of the association between selective attention and brain responses during consumption may give us more insight into how product labels can affect the consumption experience.

Previously, effects of selective attention on brain activation induced by food viewing and tasting have been studied via complex cognitive manipulations such as words, symbols or labels emphasising either the taste, caloric value or health aspects of a food cue (Grabenhorst et al. 2008; Grabenhorst et al. 2013; Linder et al. 2010; Ng et al. 2011; Nitschke et al. 2006). These manipulations were shown to modulate brain activation in reward-related regions such as the OFC, ACC, amygdala and ventral striatum. These cognitive manipulations influence specific aspects of the taste experience such as the perceived intensity, healthiness or caloric value. Nevertheless, only one study explicitly investigated and compared the effect of selective attention on two of these dimensions, namely intensity and pleasantness (Grabenhorst and Rolls 2008). They found that when participants focussed their attention on intensity, taste activation was greater in the insular cortex, but when they focussed on pleasantness, the medial orbitofrontal cortex (OFC) and anterior cingulate cortex (ACC) were more responsive during tasting a monosodium glutamate solution. In line with this, the anterior insula and overlying operculum, but not the OFC, showed greater activation when participants were instructed to detect a taste in a tasteless solution, in comparison to passive tasting (Veldhuizen et al. 2007). These studies show that taste activation in the insular cortex, OFC and ACC can be altered by selective attention. However, more research is needed to further elucidate how selective attention to specific taste aspects influences the consumption experience. In addition, it is of interest to extend this from simple solutions to regular liquid foods, which provide more than just gustatory stimulation.

Neural processing of taste intensity and valence has been linked to specific brain regions. The insula and overlying frontal operculum (which contain the primary taste cortex (Lundström et al. 2011)), are believed to represent taste intensity (Dalenberg et al. 2015; Small 2010; Small et al. 2003a; Spetter et al. 2010). Beside intensity, the primary taste cortex also represents taste quality and valence (Dalenberg et al. 2015; Small 2010). Food valence is believed to be represented in the OFC, an area that receives neural signals directly from the primary taste cortex and has been designated as secondary taste cortex (Kringelbach et al. 2003; Lundström et al. 2011; E. T. Rolls 2008; E. Rolls 2004; Small et al. 2003a. The OFC projects to the striatum and ACC (Lundström et al. 2011), which are involved in processing affective value and taste intensity (Delgado 2007; E. T. Rolls 2008; Sescousse et al. 2013; Small et al. 2003a; Spetter et al. 2010). In addition, the primary taste cortex and OFC project to the amygdala, a region possibly involved in integrating affect and intensity (Anderson et al. 2003; Baxter and Murray 2002; Small 2006; Small et al. 2003a; Spetter et al. 2010). Recently, the presence of calories in the mouth has also been associated with activation in several brain regions including the amygdala, striatum, ACC and insula and overlying frontal operculum (Chambers et al. 2009; Frank et al. 2008; Griffioen-Roose et al. 2013; Smeets et al. 2011; van Rijn et al. 2015; van Rijn et al. 2016). However, selective attention to caloric content of food in the mouth has not been investigated to our knowledge. The aim of the current study was to investigate the effect of selective attention to hedonics, intensity and caloric content on the brain activation during tasting of regular drinks. This may affect brain activation during tasting in the above listed regions that have been associated with taste intensity, valence and caloric content. Secondary, we assessed the association between brain activation during selective attention while tasting and subjective pleasantness, intensity and caloric content ratings.

## Materials and methods

### Participants

Thirty young, healthy, right-handed females with a normal weight were included in the study. One participant dropped out because of feelings of discomfort in the scanner. Furthermore, due to technical issues with the gustometer, data was not reliable for two of the subjects. Therefore, twenty-seven participants with a mean (± SD) age of 22 (± 3) y and a mean (± SD) BMI of 21.5 (± 1.7) kg/m^2^ were included in the analyses. Exclusion criteria were: a restrained eating score higher than 3.40 (Dutch Eating Behavior Questionnaire (Van Strien 2005)), an energy restricted diet during the past two months, change in body weight of more than five kg during the past two months, lack of appetite, stomach of bowel diseases, chronic diseases such as diabetes, thyroid- or kidney disease, having a history of neurological disorders, having a mental illness, use of daily medication other than oral contraceptives or paracetamol, having difficulties with swallowing and/or eating, having taste or smell disorders, being allergic and/or intolerant for products under study, smoking more than one cigarette/cigar a day, having a history of or current alcohol consumption of more than 21 units per week, being pregnant or lactating, having any contra-indication for MRI scanning or disliking the product under study (liking <5 on a 9-point scale). Before enrollment, participants were screened on inclusion and exclusion criteria via a questionnaire and a taste test. After screening, included participants completed a training session in which they practiced the fMRI procedure. All participants gave written informed consent. This study was conducted in accordance with the Declaration of Helsinki (amendment of Fortaleza, 2013), approved by the Medical Ethical Committee of Wageningen University and registered in the Dutch Trial Registry (NTR5253).

### Stimuli

Stimuli consisted of a commercially available fruit juice (Dubbel Drank orange and peach, Appelsientje, 48 kcal/100 mL, Royal FrieslandCampina, Amersfoort, The Netherlands), tomato juice (Zontomaat, Appelsientje, 18 kcal/100 mL, Royal FrieslandCampina, Amersfoort, The Netherlands) and tap water, administered at room temperature.

### Experimental procedure

**Fig. 1:**
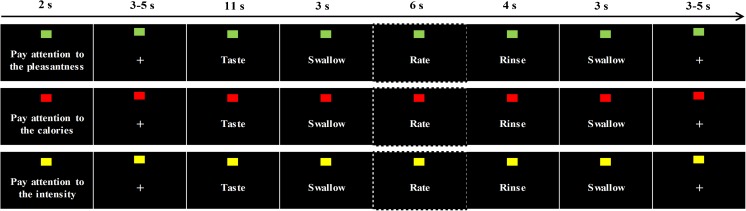
Schematic overview of trial structures during a block of the taste-task

Participants arrived between 08:00 and 10:00 h at the test location (Hospital Gelderse Vallei, Ede, The Netherlands) after a fast of at least 3 h (no food, only water) and were placed into the MRI scanner to engage in an fMRI taste-task. During this task, participants tasted small sips (2 mL) of the fruit juice, tomato juice and water while they had been instructed to pay attention to either the pleasantness, taste intensity or amount of calories of the stimulus. Participants were led to believe that they were tasting two types of fruit juice and two types of tomato juice. They were told that the two fruit juices and the two tomato juices were very similar tasting, but that there were slight differences in ingredients. The task consisted of three runs and one run consisted of three blocks: a pleasantness block, an intensity block and a calorie block. Figure 1 shows a schematic overview of the trial structures during a block. At the beginning of each block, a screen that indicated to which characteristic participants had to pay attention was shown (block cue, based on Hare et al. 2011). This was indicated in words (pay attention to the pleasantness, calories or taste intensity), as well as with the color of a square that was depicted on the top of the screen. This colored square was present during the whole task and changed color at the start of a new block. Moreover, beforehand, participants also had been asked to memorize the three color-instruction combinations. The order of the blocks varied during the runs and the order of the runs varied between participants. During each block, every stimulus was tasted 4 times. Within blocks the order of stimulus administration was pseudorandom. This resulted in 12 trials per characteristic per stimulus in total. A trial consisted of a 11-s taste-event, followed by a 3-s swallow, a 4-s rinse with water, a 3-s swallow and a 3–5-s rest. During each block, participants rated either the pleasantness, taste intensity or amount of calories congruent with the type of block, once for each stimulus on a 5-point scale, anchored with ‘not at all’ till ‘very’, or for calories, ‘none’ till ‘very much’. Ratings were given after the 2nd or 3rd administration of each stimulus, directly after swallowing. Instructions to either taste, swallow, rate, rinse or rest were given to participants via visual cues on a screen placed in the bore at the back end of the scanner. Stimuli were administered with the use of programmable syringe pumps (New Era Pump Systems Inc., Wantagh, NY) at 50 mL/min.

### MRI data acquisition

A scan session consisted of 3 functional runs during which 460 functional volumes were acquired using a T_2_^*^-weighted gradient echoplanar imaging sequence (TR = 2140 ms, TE = 25 ms, 90° flip angle, FOV = 192 × 192 mm, 43 axial slices, descending order, voxel size 3 × 3 × 3 mm) on a 3 T Siemens Magnetom Verio (Siemens, Erlangen, Germany). The stack was tilted at an angle of 30° to the anterior-posterior commissure line to reduce signal dropout in orbitofrontal cortex and ventral temporal lobe (Deichmann et al. 2003). Additionally, a high-resolution T_1_-weighted anatomical scan was acquired (MPRAGE, TR = 2300 ms, TE = 2.98 ms, 9° flip angle, FOV = 256 × 256 mm, 192 sagittal slices, voxel size =1 × 1 × 1 mm).

### Data analysis

fMRI data were preprocessed and analyzed with the SPM8 software package (Wellcome Department of Imaging Neuro-science, London, UK) in conjunction with the MarsBar toolbox (http://marsbar.sourceforge.net/) run with MATLAB 7.12 (The Mathworks Inc., Natick, MA). The functional volumes of every participant were slice time corrected, realigned to the first volume of the first run, coregistered to the anatomical image, globally normalized to the Montreal Neurological Institute space (MNI space), and spatially smoothed with a Gaussian kernel of 6 mm full-width at half-maximum. A statistical parametric map was generated for every participant by fitting a boxcar function to each time series, convolved with the canonical hemodynamic response function. Data were high-pass filtered with a cutoff of 128 s. For each taste stimulus, 3 conditions of interest were modelled: paying attention to intensity, caloric content and pleasantness. Furthermore, 4 conditions of no interest were modelled: rinsing, swallowing, task instructions and rating. To account for motion-related variance, realignment parameters were added to the model as regressors of no interest. For every participant, parameters were estimated for the intensity, calorie and pleasantness conditions by averaging over fruit juice, tomato juice and water (versus baseline) in T-contrasts. Brain responses were averaged over the stimuli to increase power and to be able to generalize over taste quality and pleasantness level (see e.g. Bender et al. 2009). Furthermore, all selective attention conditions were contrasted against each other using T-contrasts. This yields the differences due to selective attention, while cancelling out common activation of no interest like somatosensory and motor activation. On the group level, region of interest (ROI) analyses were performed. A priori ROIs were areas associated with taste processing: the OFC, insula, frontal and rolandic operculum, ACC, amygdala, caudate, putamen, pallidum and thalamus (Lundström et al. 2011; Sescousse et al. 2013; Small 2006; Veldhuizen et al. 2011). A combined mask of these regions was created with the WFU Pickatlas tool (Maldjian et al. 2003) and used in ROI analyses with small volume correction over the mask volume.

First, common activation for the selective attention conditions in the ROIs was examined by means of a conjunction analysis. A one-way within-subject ANOVA was performed using the subject-level contrasts for each selective attention condition versus baseline to create a model with the three selective attention conditions as levels. Hereafter, separate T-maps were created for the selective attention conditions and these were combined into a conjunction T-map (conjunction null). The resulting conjunction T-map was thresholded at *T* = 8 and a cluster size of k > 4 contiguous voxels.

Second, differences in selective attention-related activation within the ROI mask were examined with three one-sample t-tests in which the subject-level contrast images for the comparisons between the attention conditions were entered. In addition, we tested for correlations between brain activation during selective attention (versus baseline) and subjective ratings within the ROI mask by means of one-sample T-tests to which the respective ratings were added as a covariate (mean-centered). Resulting T-maps were thresholded at *P* < 0.001 (uncorrected for multiple comparisons) and a cluster size of k > 4 contiguous voxels. This threshold is based on Lieberman and Cunningham (2009), who argue for less conservative thresholding and even advise a less stringent threshold of *P* < 0.005 with a 10 voxel cluster extent. Too conservative thresholding in an attempt to decrease false positive effect (type I errors), may increase the possibility for missing true effects (Type II errors), and may introduce biases toward studying large rather than small effects and observing sensory and motor processes rather than complex cognitive and affective processes (Lieberman and Cunningham 2009). For visualization of the correlations average parameter estimates for each cluster were extracted with the use of the MarsBar toolbox.

Subjective ratings were analyzed with SPSS. The different ratings for each stimulus were averaged over the three runs. Subsequently, differences between the stimuli were tested for using repeated measures ANOVA in conjunction with post-hoc T-tests, *P* < 0.05, LSD-corrected for multiple comparisons.

## Results

### Subjective ratings

**Fig. 2:**
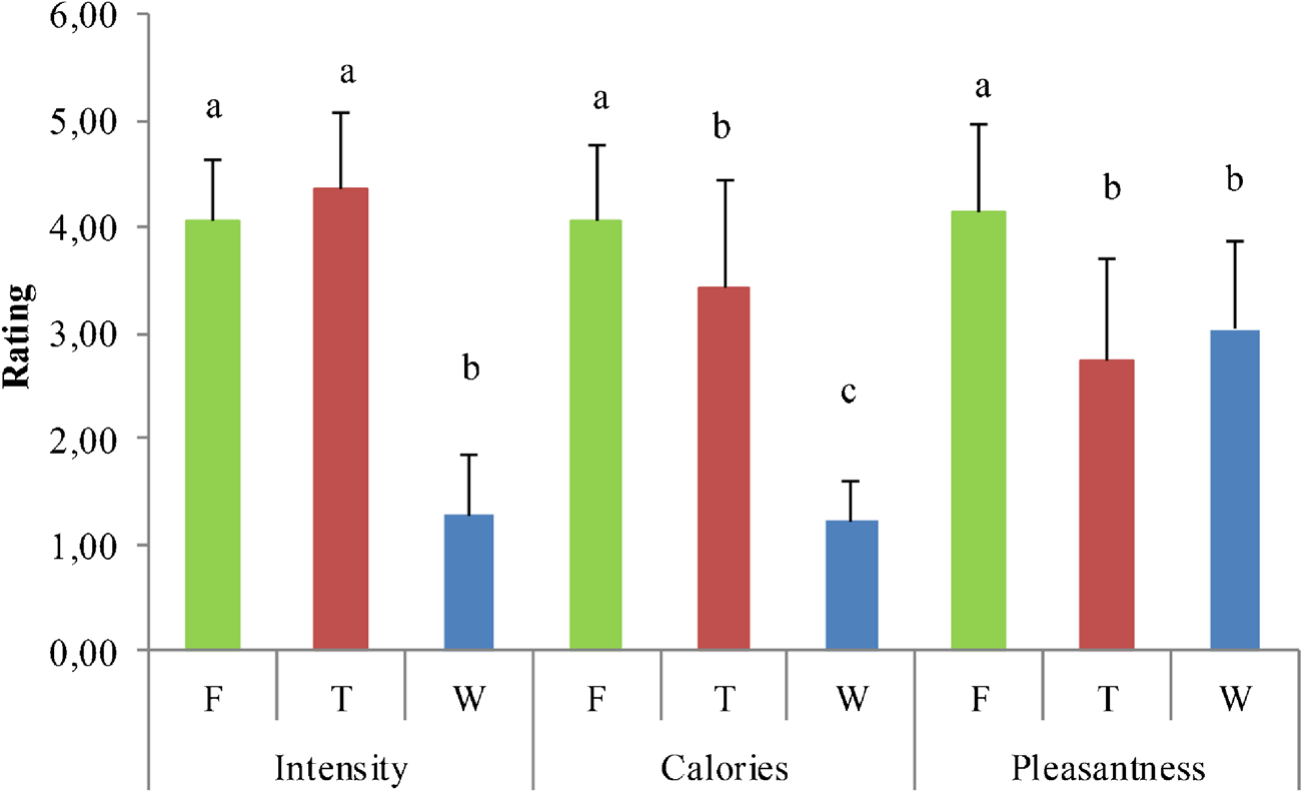
Subjective ratings (mean + SD) on a 5-point scale for intensity during selective attention to intensity, caloric content during selective attention to caloric content and pleasantness during selective attention to pleasantness of a fruit juice (F), tomato juice (T) and water (W), obtained during scanning. Repeated measures ANOVA on average ratings over the three runs, post-hoc t-tests, *P* < 0.05, LSD-corrected for multiple comparisons. Bars within each condition that have a different letter differ significantly

Subjective ratings for intensity, caloric content and pleasantness of the fruit juice, tomato juice and water, obtained during scanning can be found in Fig. 2. Water was significantly less intense than the juices. Furthermore, fruit juice was perceived as most calorie dense, followed by tomato juice and water. Finally, fruit juice was perceived as most pleasant.

### Common brain activation during selective attention while tasting

**Fig. 3:**
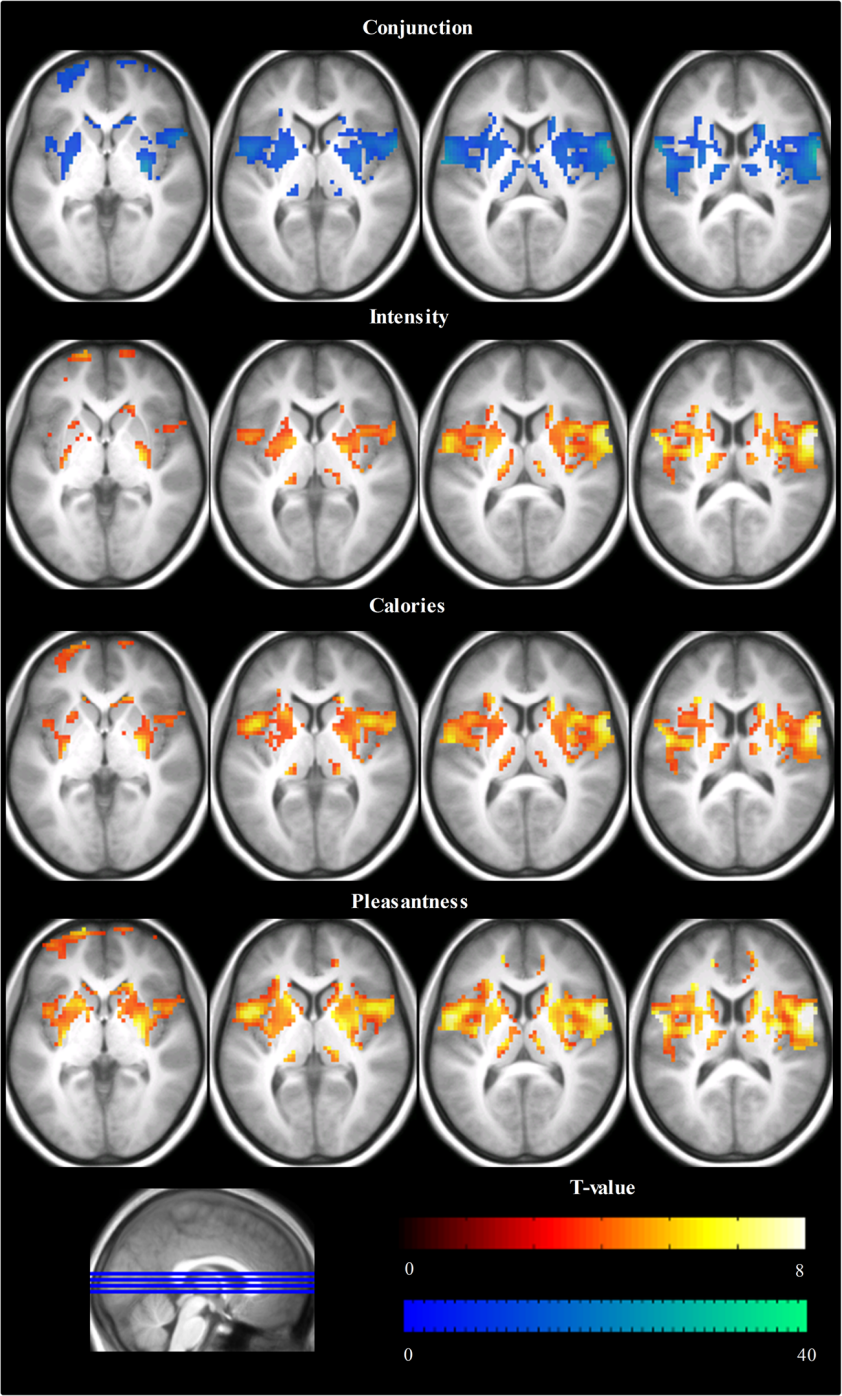
Illustration of Brain activation versus rest during selective attention to taste intensity, caloric content and pleasantness (thresholded at *P* = 0.001, k = 4) and their conjunction (Top, thresholded at *T* = 8, k = 4). Shown are T-maps in axial sections at MNI z-coordinates −1, 4, 9 and 14). Full details can be found in Supplementary Table 1- 4

Figure 3 shows the brain responses during selective attention to the intensity, caloric content or pleasantness while tasting and the conjunction for these selective attention conditions (also see Supplementary Tables 1, 2, 3 and 4). Common brain activation was observed in the rolandic operculum, insula and overlying frontal operculum, striatum, amygdala, thalamus, anterior cingulate cortex and middle OFC.

### Differential effects of selective attention on brain activation during tasting

**Fig. 4:**
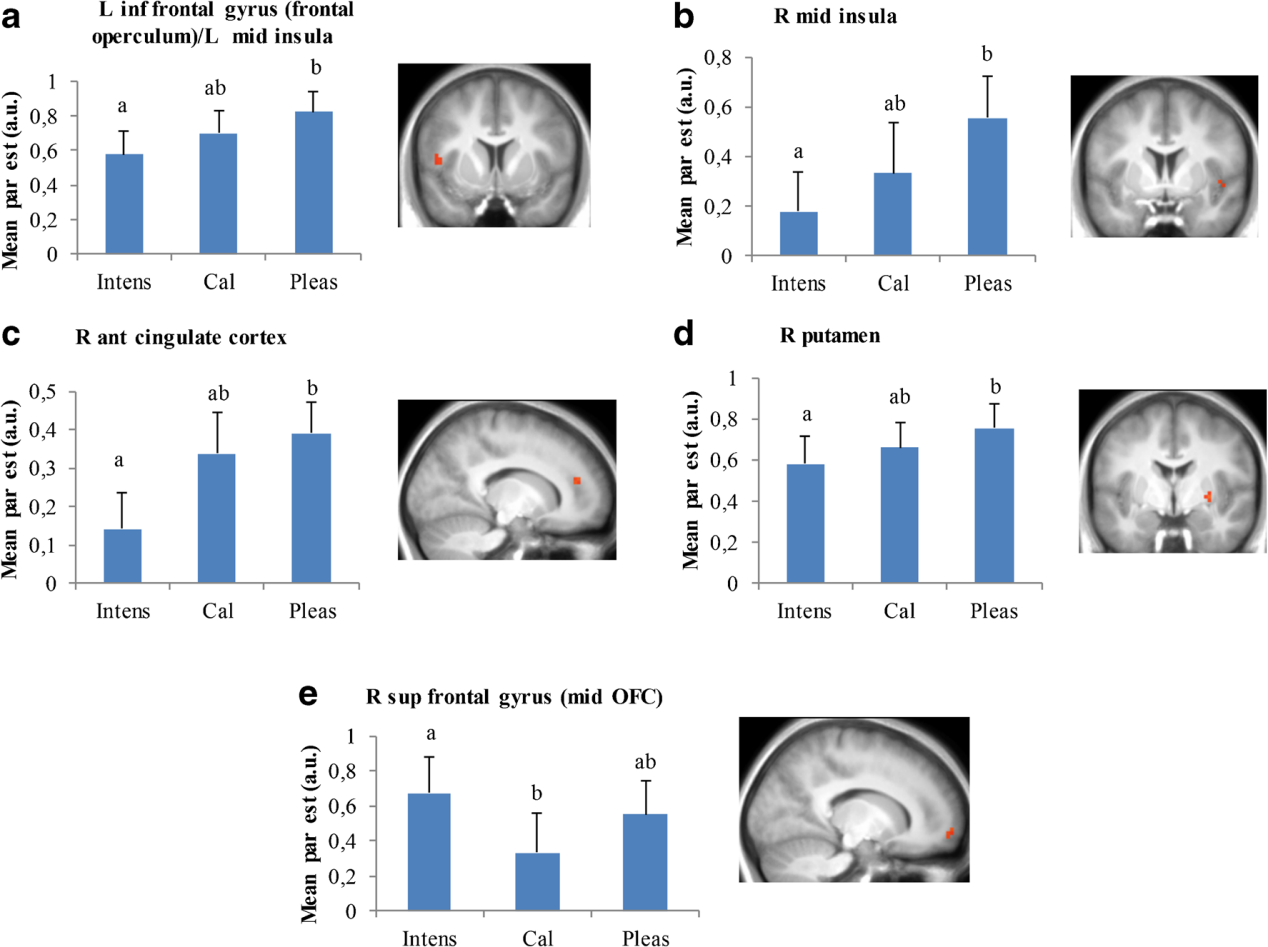
Illustration of differences in brain activation during selective attention to taste intensity (intens), caloric content (cal) and pleasantness (pleas) in the (**a**) left inferior frontal gyrus (frontal operculum)/left middle insula (peak at MNI: −45, 11, 4), (**b**) right mid insula (peak at MNI: 45, 2, −2), (**c**) right anterior cingulate cortex (peak at MNI: 15, 44, 19), (**d**) right putamen (peak at MNI: 30, −4, −2), and (**e**) right superior frontal gyrus (mid OFC) (peak at MNI: 15, 62, −5). Shown are average cluster parameter estimates (+SE) for the contrast versus rest, obtained for the clusters showing significant differences between conditions, as assessed with one-sample t-tests on the respective contrasts (*P* < 0.001, k > 4, see Table 1). Bars having a different letter differ significantly. Ant = anterior, sup = superior, inf = inferior, mid = middle, L = left and R = right

Table 1 shows the differences in brain activation between the three selective attention conditions (also see Fig. 4). Attentional focus on intensity resulted in more activation in the right middle OFC compared to when attention was directed to caloric content. Paying attention to the pleasantness compared with intensity, induced more activation in the right and left middle insula, the left frontal operculum, right ACC and right putamen.

**Table 1:** Differences in brain activation during tasting while paying attention to intensity, calories or pleasantness

Comparison	Brain region	Cluster size	Z-score	Peak coordinate		
				x	y	z
Intensity - Calories	R sup frontal gyrus (mid OFC)	7	3.4	15	62	-5
Calories - Intensity	No regions					
Intensity - Pleasantness	No regions					
Pleasantness - Intensity	R putamen	5	3.5	30	-4	-2
	R ant cingulate cortex	5	3.4	15	44	19
	R mid insula	5	3.2	45	2	-2
			3.2	42	-1	-5
	L inf frontal gyrus (frontal operculum)/extending into L mid insula	8	3.2	-45	11	4
Calories - Pleasantness	No regions					
Pleasantness - Calories	No regions					

Activations were thresholded at *p* < 0.001, with small volume correction over the ROI volume and a cluster extent threshold of k > 4 contiguous voxels. Ant = anterior, sup = superior, inf = inferior, mid = middle, L = left and R = right

### Relationship between brain activation during attentional focus on intensity, calories or pleasantness and subjective ratings

**Fig. 5:**
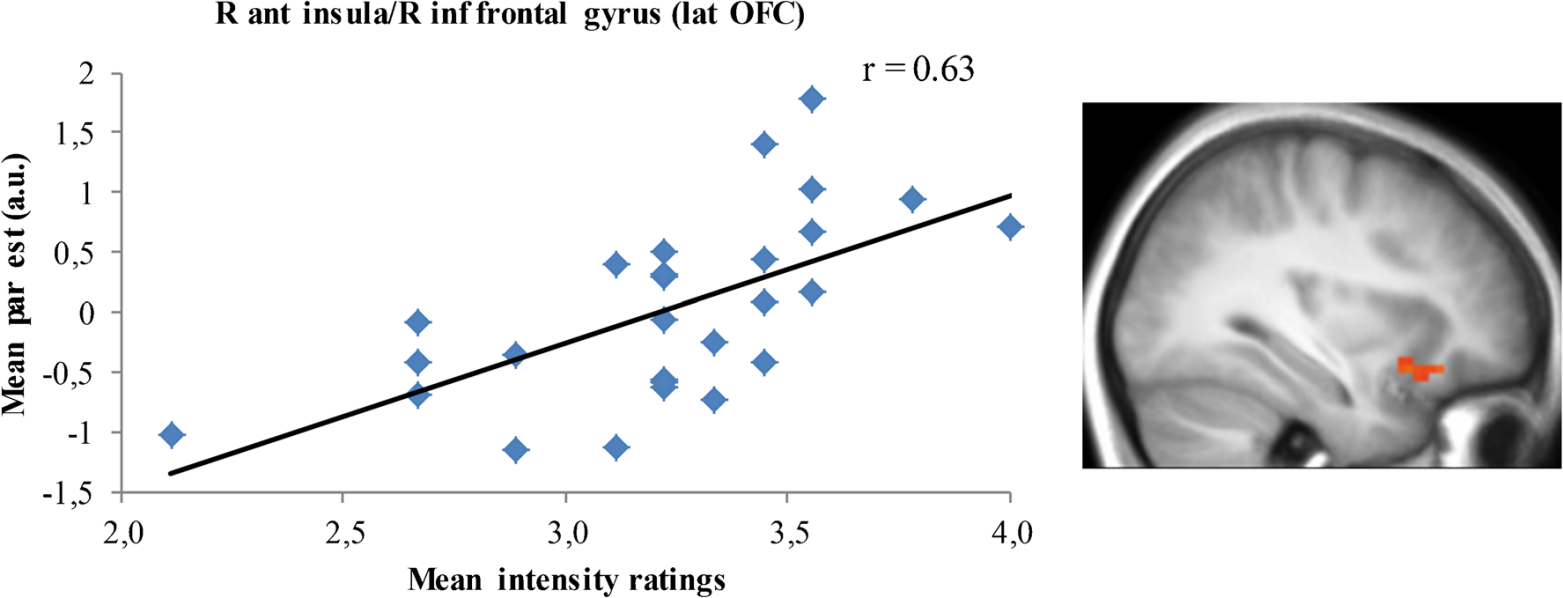
Scatterplot illustrating the correlation between brain activation during selective attention to taste intensity and subjective intensity ratings in a cluster in the right anterior insula extending into the right inferior frontal gyrus (lateral OFC) (peaks at MNI: 33 17–14 and 33 29–14, see Table 2). Ant = anterior, inf = inferior, lat = lateral and R = right

Table 2 shows brain regions in which an association was found between subjective ratings and brain activation in the three selective attention conditions. There was a positive correlation between intensity ratings and activation during attentional focus on taste intensity in the right anterior insula and right lateral OFC (Fig. 5). No correlations were found between calorie and pleasantness ratings and activation during attentional focus on respectively calories and pleasantness of the drinks.

**Table 2:** Brain regions in which there was a significant positive correlation between subjective ratings and brain activation during tasting in the three selective attention conditions

Correlation	Brain region	Cluster size	Z-score	Peak coordinate		
				x	y	z
Intensity ratings (in intensity condition)	R ant insula	18	3.5	33	17	-14
	R inf frontal gyrus (lat OFC)		3.4	33	29	-14
Calorie ratings (in calorie condition)	No regions					
Pleasantness ratings (in pleasantness condition)	No regions					

Activations were thresholded at *p* < 0.001, with small volume correction over the ROI volume and a cluster extent threshold of k > 4 contiguous voxels. Ant = anterior, inf = inferior, lat = lateral and R = right

## Discussion

We investigated the effect of selective attention to hedonics, intensity and caloric content on brain responses during tasting. Brain activation for these selective attention conditions largely overlapped; common activation was found in regions associated with taste processing and food reward such as the rolandic operculum, insula and overlying frontal operculum, striatum, amygdala, thalamus, ACC and middle OFC. Brain activation during selective attention to taste intensity compared to calories was higher in the right middle OFC. Furthermore, brain activation during selective attention to taste pleasantness compared to intensity was greater in the right putamen, right ACC and bilateral middle insula, areas associated with food reward. In addition, there was a positive association between brain activation during selective attention to taste intensity and intensity ratings in the right anterior insula and right lateral OFC.

### Common brain activation during selective attention while tasting

There was substantial overlap in brain activation while tasting during selective attention to intensity, caloric value and pleasantness in regions involved in the neural processing of food stimuli. This may be expected because ‘bottom-up’ effects are equal for all selective attention conditions and are prominent in comparisons versus low-level baseline (fixation on a crosshair). This is in line with a study with a similar paradigm, in which taste activation overlapped greatly in the anterior insula and overlying frontal operculum irrespective of whether participants had to indicate the quality, the presence or the pleasantness of a taste, or just tasted passively (Bender et al. 2009). Cognitive effects, such as selective attention effects, are often more difficult to detect with neuroimaging than motor or sensory effects due to greater trial-to-trial and person-to-person variation (Griffin and Ross 1991; Lieberman and Cunningham 2009) and because they are more subtle. This could be the reason that we found relatively few effects of selective attention.

### Differential effects of selective attention on brain activation during tasting

#### Insula

Selective attention to pleasantness versus intensity but not caloric content, induced more activation in the bilateral middle insula and left overlying frontal operculum. The middle insula has been implicated in the detection of actual intensity differences (Small et al. 2003a; Spetter et al. 2010). However, recently Dalenberg et al. (2015) examined the functional specialization of the insula regarding taste processing in more detail and found that activation in the middle insula is related to the presence of a taste and it’s corresponding pleasantness (Dalenberg et al. 2015). Several other studies are consistent with a role for the middle insula in pleasantness coding (Bender et al. 2009; de Araujo et al. 2003; Pelchat et al. 2004). For example, Bender et al. (2009) reported that attending to the pleasantness of a taste produced large responses in the middle insula. De Araujo et al. (2003) found that water in the mouth activated the middle insula, but only when participants were thirsty, and thus perceived the water as more pleasant. Finally, Pelchat et al. (2004) found that imagining to eat a pleasant food in comparison to a bland food resulted in increased middle insula activation for participants who had been consuming a monotonous diet for 1.5 days. This latter study agrees with ours that an attentional focus on pleasantness increases middle insula activation. Furthermore, we found that intensity ratings correlated with taste activation during selective attention to intensity in the right anterior insula. This is concurrent with Grabenhorst and Rolls (2008) and Dalenberg and colleagues’ finding that right anterior insula activation is associated with concentration rather than pleasantness (Dalenberg et al. 2015). Overall, our results strengthen the idea that the middle insula is involved in pleasantness coding and the anterior insula in intensity coding.

#### OFC

Compared to caloric content, both focussing on intensity and pleasantness during tasting activated the middle OFC more (statistically significant for intensity, trend for pleasantness). Tang et al. (2014) showed that humans are poor at estimating the caloric content of food on pictures. Estimating caloric content may be a largely unconscious process and thus less accessible to conscious evaluations. For example, actual, rather than estimated caloric density of food pictures, predicts the willingness to pay for a food item (Tang et al. 2014). Furthermore, estimated expected satiety of food images is not in line with their actual energy content (Brunstrom et al. 2008). Evaluating calories based on oral sensations rather than on food pictures, as was done in the current study, may even be more difficult. Participants may have focussed on sensory cues associated with caloric content such as sweetness, viscosity and creaminess to try and detect caloric content, but in reality there were no differences in caloric content for the same stimulus type and clear differences between the three stimuli in the sensory cues associated with calories. Therefore, they may also have tried to ‘search’ for calories otherwise. This is not something that people would normally do, or be asked to do. Thus, we speculate that the other two attention conditions may have elicited a better attentional focus; evaluating hedonic value is commonly done and evaluating stimulus intensity is something that we know people can do. This could explain why consciously focussing on calories did not elicit greater activation compared to focussing on the other product properties in any of the ROIs.

No differences between attentional focus to intensity versus pleasantness were found in the OFC. Also, we did not find a correlation between pleasantness ratings and medial OFC activation during attending pleasantness, which was expected based on Grabenhorst and Rolls (2008). The medial parts of the OFC are involved in decoding and monitoring reward, whereas the lateral parts are involved in evaluating punishment (Kringelbach and Rolls 2004; O’Doherty et al. 2001). Therefore, evaluating the pleasantness of the stimuli was expected to result in the most prominent activation in this area. However, taste intensity and pleasantness are not independent: in general their relationship can be captured in an inverted U-shaped curve (Veldhuizen et al. 2006). For salty and sour stimuli, intensity and pleasantness are positively correlated up to the peak, whereupon pleasantness declines. Sweetness is almost increasingly pleasant with increasing intensity (Pfaffmann 1980). It is therefore difficult to disentangle brain regions involved in encoding pleasantness and intensity (Dalenberg et al. 2015; Spetter et al. 2010). Thus, brain activation in the OFC during tasting may not only dependent on pleasantness, but also on intensity. Based on our results, this holds true for the lateral OFC where we observed a positive association between brain activation when focussing on taste intensity and intensity ratings. Nevertheless, several other studies have been able to show pleasantness representation in the OFC (e.g. Grabenhorst and Rolls 2008; Kringelbach et al. 2003). Thus, this warrants further research into the representation of hedonic value in both the OFC and insula (e.g. Dalenberg et al. 2015) and the experimental conditions under which this can be measured with fMRI.

#### Putamen

Attentional focus to pleasantness compared to intensity resulted in more taste activation in the putamen, a part of the dorsal striatum (Delgado 2007). Taste activation in the putamen was found to be modulated by sweetness, saltiness and bitterness irrespective of valence, (Small et al. 2003a; M S Spetter et al. 2010), implying its involvement in intensity processing. However, others found that the dorsal striatum is involved in coding food reward (Berridge 1996; Pelchat et al. 2004; Small et al. 2003b; Stoeckel et al. 2008). Especially reward receipt, rather that reward anticipation, is processed by the dorsal striatum (Small 2006). Our findings are consistent with a role for the dorsal striatum in reward receipt, and additionally suggest that selective attention to hedonics can enhance taste-related activation in this region.

#### ACC

The ACC is implicated in reward receipt (Small 2006). We found that selective attention to taste pleasantness in comparison to intensity was associated with increased ACC activation. The exact location of our finding is in the dorsal (also referred to as posterior) part of the ACC (Brodmann area 32). This specific part has been labelled as the ‘cognitive division’ and is activated by cognitive rather than by emotionally demanding tasks (Bush et al. 2000). In agreement with our finding, Grabenhorst and Rolls (2008) also observed increased responses in the ACC during paying attention to pleasantness compared to intensity of a monosodium glutamate (umami) solution. We show that this generalizes over taste qualities (sweet, savory and neutral liquids).

### Selective attention in functional neuroimaging taste paradigms

Across functional neuroimaging studies, differences in selective attention can be introduced by the different participant instructions used during the delivery of taste stimuli in the broad sense of the word (both pure gustatory stimuli and more complex liquid foods), such as the words: ‘taste’, ‘test’ or ‘hold the solution in your mouth’, a colored field or just a crosshair on the screen (Felsted et al. 2010; Grabenhorst et al. 2010; Iannilli et al. 2012; Kerr et al. 2014; Schoenfeld et al. 2004; Spetter et al. 2010; van den Bosch et al. 2014). As a result, participant attention is directed in many different ways, which could lead to variability in taste activation within and between studies, as the elegant study of Grabenhorst and Rolls (2008) and Bender et al. (2009) have demonstrated with the use of pure gustatory stimuli and explicit attentional focus instructions. Accordingly, we observed that selective attention to different food properties can indeed result in differences in brain activation during tasting, also for regular drinks. During neuroimaging taste research, selective attention may therefore act as a confounding factor. In general, reproducibility of (food-related) neuroimaging findings is often difficult (Button et al. 2013; van Meer et al. 2015; van der Laan et al. 2011). However, for taste processing there is good consensus on the core regions involved (Kringelbach et al. 2004; Veldhuizen et al. 2011; Lundström et al. 2011). Nevertheless, our results confirm that selective attention biases, introduced by a variety of participant instructions, can affect taste-related neuroimaging findings.

### Strengths and limitations

A novel aspect of this study compared to previous work (Grabenhorst and Rolls 2008; Bender et al. 2009) is the use of a sweet and savoury drink, which provide flavour stimulation, rather than pure gustatory stimuli. Also, we asked participants to attend to calorie content in addition to taste intensity and pleasantness. This might be employed also in future studies in potentially more caloric drinks like yoghurt drinks or milk or protein shakes. A notable difference in the fMRI design was that we used a block design, with one attentional instruction for a series of taste trials (based on Hare et al. 2011) whereas previous studies used a separate attentional instruction for each trial (Grabenhorst and Rolls 2008; Bender et al. 2009). The latter may yield more pronounced selective attention effects, but it may also be more demanding for participants to switch attentional focus so often. The drawback of our approach is that it prevented us from doing a parametric modulation analysis with the subjective ratings on a trial-by-trial basis. However, this would have resulted in a much longer paradigm. For the same reason we did not include a ‘no attention’ control condition which, however, would have provided more information on the brain activity during attention to calories. Such a control condition could also have verified our assumption that in the absence of a specific attention instruction people tend to attend to the hedonic value of foods. We also note that it is not possible to verify attentional focus during tasting, other than by explicitly asking. Since we lack a behavioral measure of attention the conclusions that can be drawn with respect to behavior are limited.

### Conclusion

Paying attention to the hedonics, caloric content or taste intensity of a liquid predominantly resulted in common brain activation in regions involved in the neural processing of food stimuli. This likely resulted from ‘bottom-up’ sensory effects, which are more prominent than ‘top-down’ attentional effects. Nevertheless, differences were observed between selective attention to intensity versus calories in the right middle OFC, and between selective attention to pleasantness versus intensity in the right putamen, right ACC and bilateral middle insula. Furthermore, intensity ratings correlated with brain activation during selective attention to taste intensity in the anterior insula and lateral OFC.

Our data suggest a role for the middle and lateral OFC and anterior insula in evaluating intensity of a stimulus rather than caloric content or pleasantness. Moreover, selective attention to pleasantness enhanced activation in regions associated with food reward, such as the putamen, ACC and middle insula. Attentional focus on caloric content did not increase brain activation while tasting in any region. This implies that explicitly evaluating caloric content is difficult for humans and that this is probably done in a more implicit manner.

In conclusion, selective attention on pleasantness, taste intensity or caloric content can alter the activation of gustatory and reward regions. This may underlie effects of food labels on the consumption experience of consumers.

## Electronic supplementary material

Supplementary Table 1 Overlapping brain activation during tasting while paying attention to the intensity, calories or pleasantness. (DOCX 22 kb)

Supplementary Table 2 Average brain activation during tasting compared to rest, while paying attention to the intensity. (DOCX 22 kb)

Supplementary Table 3 Average brain activation during tasting compared to rest, while paying attention to the calories. (DOCX 22 kb)

Supplementary Table 4 Average brain activation during tasting compared to rest, while paying attention to the pleasantness. (DOCX 24 kb)
