# Supplementary material for: It’s in the eye of the beholder: selective attention to drink properties during tasting influences brain activation in gustatory and reward regions
Source: Brain Imaging Behav. 2017 Mar 20;12(2):425–36. doi: 10.1007/s11682-017-9710-2 (PMC5880857; doi:10.1007/s11682-017-9710-2)
Supplement: Supplementary file 1 — Overlapping brain activation during tasting while paying attention to the intensity, calories or pleasantness. (DOCX 22 kb) [file 11682_2017_9710_MOESM1_ESM.docx]

**Supplementary Table 1**

|  |  |  | |  |  |  |  |
| --- | --- | --- | --- | --- | --- | --- | --- |
| ***Conjunction*** | ***Brain region*** | ***Cluster size*** | | ***Z-score*** | *Peak coordinate* | | |
|  |  |  |  |  | ***x*** | ***y*** | ***z*** |
|  |  |  | |  |  |  |  |
| **Intensity, calories and pleasantness** | R rolandic operculum | 1072 | | Infinity | 60 | -4 | 16 |
|  |  |  | | Infinity | 54 | -7 | 19 |
|  |  |  | | Infinity | 60 | 2 | 16 |
|  |  |  | | Infinity | 57 | -16 | 16 |
|  |  |  | | Infinity | 39 | -31 | 16 |
|  |  |  | | Infinity | 54 | 11 | -2 |
|  | R caudate |  | | Infinity | 21 | 5 | 22 |
|  |  |  | | Infinity | 21 | 17 | 16 |
|  |  |  | | Infinity | 15 | 26 | -5 |
|  |  |  | | Infinity | 21 | 26 | 10 |
|  | R insula |  | | Infinity | 33 | -10 | 16 |
|  |  |  | | Infinity | 42 | 8 | 7 |
|  | R putamen |  | | Infinity | 30 | -10 | -2 |
|  |  |  | | Infinity | 30 | -7 | -8 |
|  |  |  | | Infinity | 27 | -1 | -8 |
|  | R amygdala |  | | Infinity | 24 | 2 | -14 |
|  | L inf frontal gyrus (frontal operculum) | 807 | | Infinity | -60 | 5 | 16 |
|  |  |  | | Infinity | -60 | 11 | 22 |
|  | L rolandic operculum |  | | Infinity | -60 | -4 | 10 |
|  |  |  | | Infinity | -48 | -13 | 22 |
|  |  |  | | Infinity | -51 | -10 | 13 |
|  |  |  | | Infinity | -51 | 2 | 4 |
|  |  |  | | Infinity | -45 | -28 | 13 |
|  |  |  | | Infinity | -51 | -22 | 13 |
|  | L caudate |  | | Infinity | -21 | 2 | 22 |
|  |  |  | | Infinity | -18 | -10 | 19 |
|  | L thalamus |  | | Infinity | -12 | -19 | 7 |
|  |  |  | | Infinity | -15 | -13 | 16 |
|  |  |  | | Infinity | -3 | -10 | 10 |
|  | L putamen |  | | Infinity | -24 | 5 | 7 |
|  |  |  | | Infinity | -24 | -4 | 13 |
|  | L insula |  | | Infinity | -36 | -10 | 16 |
|  | L ant cingulate cortex | 76 | | Infinity | -9 | 14 | 28 |
|  |  |  | | Infinity | -6 | 5 | 28 |
|  |  |  | | Infinity | 0 | 2 | 28 |
|  | R ant cingulate cortex |  | | Infinity | 9 | 17 | 28 |
|  | L sup frontal gyrus (mid OFC) | 87 | | Infinity | -21 | 62 | -5 |
|  |  |  | | 7.8 | -21 | 47 | -11 |
|  |  |  | | 7.6 | -18 | 44 | -14 |
|  | L mid frontal gyrus (mid OFC) | | | 7.4 | -30 | 47 | -2 |
|  |  | |  | 7.4 | -21 | 38 | -14 |
|  | R mid frontal gyrus (mid OFC) | | 36 | Infinity | 33 | 59 | -8 |
|  | R sup frontal gyrus (mid OFC) | | | Infinity | 30 | 62 | -5 |
|  | R med frontal gyrus (mid OFC) | | | 7.5 | 12 | 68 | -2 |
|  | R thalamus | | 9 | Infinity | 15 | -31 | 1 |
|  |  | |  | 7.3 | 18 | -31 | 7 |
|  | L caudate | | 8 | 7.7 | -12 | 23 | -5 |
|  |  | |  | 7.7 | -15 | 26 | -2 |
|  |  | |  | 7.2 | -6 | 20 | -2 |
|  | R sup frontal gyrus (mid OFC) | | 5 | 7.3 | 24 | 41 | -14 |
|  | L inf frontal gyrus (frontal operculum) | | 9 | 6.6 | -45 | 20 | 34 |
|  |  | |  |  |  |  |  |

Activations were thresholded at t = 8, with small volume correction over the ROI volume and a cluster extent threshold of k>4 contiguous voxels. Ant = anterior, sup = superior, inf = inferior, mid = middle, med = median, L = left and R = right.
