# Supplementary material for: It’s in the eye of the beholder: selective attention to drink properties during tasting influences brain activation in gustatory and reward regions
Source: Brain Imaging Behav. 2017 Mar 20;12(2):425–36. doi: 10.1007/s11682-017-9710-2 (PMC5880857; doi:10.1007/s11682-017-9710-2)
Supplement: Supplementary file 2 — Average brain activation during tasting compared to rest, while paying attention to the intensity. (DOCX 22 kb) [file 11682_2017_9710_MOESM2_ESM.docx]

**Supplementary Table 2**

|  |  |  |  |  |  |  |
| --- | --- | --- | --- | --- | --- | --- |
| ***Contrast*** | ***Brain region*** | ***Cluster size*** | ***Z-score*** | *Peak coordinate* | | |
|  |  |  |  | ***x*** | ***y*** | ***z*** |
|  |  |  |  |  |  |  |
| **Intensity** | R rolandic operculum | 913 | 6.1 | 60 | 2 | 13 |
|  | R rolandic operculum |  | 5.7 | 54 | -10 | 19 |
|  | R insula |  | 5.6 | 33 | -4 | 16 |
|  | R rolandic operculum |  | 5.4 | 39 | -34 | 22 |
|  | R pallidum |  | 4.9 | 27 | -7 | -5 |
|  | R pallidum |  | 4.8 | 27 | -13 | -2 |
|  | R caudate |  | 4.6 | 21 | 2 | 22 |
|  | R putamen |  | 4.5 | 21 | 17 | 13 |
|  | R inf frontal gyrus (frontal operculum) |  | 4.5 | 42 | 11 | 10 |
|  | R insula |  | 4.5 | 36 | 8 | 10 |
|  | R thalamus |  | 4.5 | 12 | -19 | 7 |
|  | R amygdala |  | 4.4 | 27 | 2 | -11 |
|  | R amygdala |  | 4.3 | 33 | -1 | -23 |
|  | R pallidum |  | 4.3 | 24 | -4 | 4 |
|  | R insula |  | 4.3 | 36 | -22 | 10 |
|  | R putamen |  | 4.2 | 27 | 8 | 13 |
|  | L rolandic operculum | 596 | 5.6 | -48 | -13 | 22 |
|  | L rolandic operculum |  | 5.3 | -57 | 2 | 13 |
|  | L insula |  | 5.2 | -33 | -7 | 16 |
|  | L insula |  | 5.0 | -36 | 11 | 16 |
|  | L rolandic operculum |  | 4.9 | -51 | -1 | 16 |
|  | L rolandic operculum |  | 4.7 | -51 | -7 | 13 |
|  | L rolandic operculum |  | 4.7 | -57 | -4 | 10 |
|  | L caudate |  | 4.6 | -21 | -1 | 19 |
|  | L putamen |  | 4.6 | -27 | -10 | 13 |
|  | L thalamus |  | 4.5 | -15 | -10 | 13 |
|  | L thalamus |  | 4.4 | -15 | -19 | 10 |
|  | L pallidum |  | 4.4 | -15 | -1 | 4 |
|  | L caudate |  | 4.3 | -18 | 17 | 16 |
|  | L rolandic operculum |  | 4.2 | -48 | 2 | 7 |
|  | L caudate |  | 4.2 | -18 | 8 | 19 |
|  | L putamen |  | 4.2 | -30 | -13 | -2 |
|  | L inf frontal gyrus (lat OFC) | 20 | 5.5 | -39 | 44 | -14 |
|  | L inf frontal gyrus (lat OFC) |  | 4.1 | -30 | 38 | -17 |
|  | R mid frontal gyrus (mid OFC) | 85 | 4.8 | 30 | 59 | -8 |
|  | R sup frontal gyrus (mid OFC) |  | 4.1 | 21 | 47 | -14 |
|  | R mid frontal gyrus (mid OFC) |  | 3.8 | 36 | 53 | -11 |
|  | R sup frontal gyrus (mid OFC) |  | 3.7 | 24 | 65 | -5 |
|  | R sup frontal gyrus (mid OFC) |  | 3.3 | 15 | 59 | -14 |
|  | L sup frontal gyrus (mid OFC) | 79 | 4.7 | -18 | 65 | -5 |
|  | L sup frontal gyrus (mid OFC) |  | 4.3 | -18 | 56 | -11 |
|  | L mid frontal gyrus (mid OFC) |  | 3.6 | -33 | 50 | -8 |
|  | L insula | 13 | 4.5 | -27 | 23 | 13 |
|  | R sup frontal gyrus (mid OFC) | 5 | 4.2 | 15 | 23 | -17 |
|  | R inf frontal gyrus (lat OFC) | 5 | 4.1 | 30 | 29 | -20 |
|  | R mid cingulate cortex | 16 | 4.0 | 12 | 17 | 28 |
|  | R mid frontal gyrus (mid OFC) | 5 | 4.0 | 33 | 41 | -14 |
|  | R mid frontal gyrus (mid OFC) |  | 3.7 | 39 | 44 | -14 |
|  | R mid frontal gyrus (mid OFC) |  | 3.4 | 45 | 47 | -14 |
|  | L ant cingulate cortex | 8 | 3.8 | -9 | 14 | 28 |
|  | L insula | 5 | 3.2 | -36 | 5 | -8 |
|  |  |  |  |  |  |  |

Activations were thresholded at p<0.001, with small volume correction over the ROI volume and a cluster extent threshold of k>4 contiguous voxels. Ant = anterior, sup = superior, inf = inferior, mid = middle, lat = lateral, L = left and R = right.
