# Supplementary material for: It’s in the eye of the beholder: selective attention to drink properties during tasting influences brain activation in gustatory and reward regions
Source: Brain Imaging Behav. 2017 Mar 20;12(2):425–36. doi: 10.1007/s11682-017-9710-2 (PMC5880857; doi:10.1007/s11682-017-9710-2)
Supplement: Supplementary file 3 — Average brain activation during tasting compared to rest, while paying attention to the calories. (DOCX 22 kb) [file 11682_2017_9710_MOESM3_ESM.docx]

**Supplementary Table 3**

|  |  |  |  |  |  |  |
| --- | --- | --- | --- | --- | --- | --- |
| ***Contrast*** | ***Brain region*** | ***Cluster size*** | ***Z-score*** | *Peak coordinate* | | |
|  |  |  |  | ***x*** | ***y*** | ***z*** |
|  |  |  |  |  |  |  |
| **Calories** | R rolandic operculum | 1062 | 6.0 | 54 | -7 | 16 |
|  | R rolandic operculum |  | 5.8 | 60 | 2 | 13 |
|  | R insula |  | 5.7 | 33 | -4 | 16 |
|  | R caudate |  | 5.1 | 21 | 26 | 7 |
|  | R pallidum |  | 5.1 | 27 | -7 | -5 |
|  | R insula |  | 5.0 | 42 | 8 | 7 |
|  | R pallidum |  | 4.9 | 27 | -13 | -2 |
|  | R insula |  | 4.9 | 36 | -31 | 19 |
|  | R caudate |  | 4.8 | 21 | 8 | 19 |
|  | R putamen |  | 4.8 | 27 | -1 | -8 |
|  | R caudate |  | 4.5 | 18 | -7 | 22 |
|  | R thalamus |  | 4.4 | 18 | -13 | 16 |
|  | R caudate |  | 4.3 | 15 | 26 | -5 |
|  | R insula |  | 4.3 | 33 | -22 | 10 |
|  | R putamen |  | 4.2 | 33 | -1 | 4 |
|  | R thalamus |  | 4.1 | 18 | -31 | 4 |
|  | L rolandic operculum | 747 | 5.6 | -48 | -10 | 19 |
|  | L rolandic operculum |  | 5.2 | -51 | -7 | 13 |
|  | L insula |  | 4.8 | -27 | 23 | 13 |
|  | L rolandic operculum |  | 4.7 | -57 | 2 | 13 |
|  | L insula |  | 4.7 | -45 | 5 | 7 |
|  | L insula |  | 4.7 | -36 | -10 | 16 |
|  | L rolandic operculum |  | 4.6 | -60 | 5 | 16 |
|  | L putamen |  | 4.5 | -30 | -13 | -2 |
|  | L putamen |  | 4.5 | -21 | 14 | 7 |
|  | L inf frontal gyrus (frontal operculum) |  | 4.5 | -36 | 5 | 22 |
|  | L thalamus |  | 4.4 | -21 | -31 | 4 |
|  | L caudate |  | 4.4 | -21 | -1 | 22 |
|  | L thalamus |  | 4.4 | -12 | -19 | 7 |
|  | L putamen |  | 4.3 | -33 | -19 | -5 |
|  | L insula |  | 4.2 | -33 | 11 | 16 |
|  | L caudate |  | 4.1 | -18 | 11 | 19 |
|  | R ant cingulate cortex | 25 | 4.6 | 12 | 17 | 28 |
|  | R ant cingulate cortex |  | 3.5 | 15 | 29 | 25 |
|  | L ant cingulate cortex | 21 | 4.4 | -9 | 14 | 25 |
|  | L ant cingulate cortex |  | 3.5 | -6 | 5 | 28 |
|  | R mid frontal gyrus (mid OFC) | 52 | 4.3 | 33 | 59 | -8 |
|  | R sup frontal gyrus (mid OFC) |  | 3.6 | 15 | 68 | -2 |
|  | R mid frontal gyrus (mid OFC) |  | 3.3 | 45 | 53 | -11 |
|  | L inf frontal gyrus (lat OFC) | 7 | 4.1 | -42 | 47 | -14 |
|  | L med frontal gyrus (mid OFC) | 54 | 4.1 | -15 | 68 | -2 |
|  | L sup frontal gyrus (mid OFC) |  | 4.0 | -18 | 65 | -5 |
|  | L sup frontal gyrus (mid OFC) |  | 3.9 | -24 | 65 | -2 |
|  | L mid frontal gyrus (mid OFC) |  | 3.6 | -33 | 44 | -5 |
|  | L mid frontal gyrus (mid OFC) |  | 3.5 | -30 | 47 | -2 |
|  | L mid frontal gyrus (mid OFC) |  | 3.4 | -36 | 53 | -5 |
|  | L caudate | 6 | 4.0 | -15 | 26 | -2 |
|  | L caudate |  | 3.7 | -12 | 23 | -5 |
|  | L rolandic operculum | 6 | 3.7 | -39 | -31 | 22 |
|  |  |  |  |  |  |  |

Activations were thresholded at p<0.001, with small volume correction over the ROI volume and a cluster extent threshold of k>4 contiguous voxels. Ant = anterior, sup = superior, inf = inferior, mid = middle, med = median, L = left and R = right.
