# Supplementary material for: It’s in the eye of the beholder: selective attention to drink properties during tasting influences brain activation in gustatory and reward regions
Source: Brain Imaging Behav. 2017 Mar 20;12(2):425–36. doi: 10.1007/s11682-017-9710-2 (PMC5880857; doi:10.1007/s11682-017-9710-2)
Supplement: Supplementary file 4 — Average brain activation during tasting compared to rest, while paying attention to the pleasantness. (DOCX 24 kb) [file 11682_2017_9710_MOESM4_ESM.docx]

**Supplementary Table 4**

|  |  |  |  |  |  |  |
| --- | --- | --- | --- | --- | --- | --- |
| ***Contrast*** | ***Brain region*** | ***Cluster size*** | ***Z-score*** | *Peak coordinate* | | |
|  |  |  |  | ***x*** | ***y*** | ***z*** |
|  |  |  |  |  |  |  |
| **Pleasantness** | R rolandic operculum | 1294 | 6.5 | 57 | 2 | 13 |
|  | R rolandic operculum |  | 6.3 | 54 | -7 | 16 |
|  | R rolandic operculum |  | 5.8 | 39 | -34 | 22 |
|  | R pallidum |  | 5.7 | 27 | -7 | -5 |
|  | R pallidum |  | 5.4 | 27 | -13 | -2 |
|  | R caudate |  | 5.4 | 21 | 8 | 16 |
|  | R putamen |  | 5.3 | 33 | -7 | 13 |
|  | R caudate |  | 5.3 | 21 | 17 | 10 |
|  | R caudate |  | 5.2 | 21 | 26 | 10 |
|  | R insula |  | 5.0 | 45 | 8 | 7 |
|  | R rolandic operculum |  | 4.9 | 45 | 2 | 22 |
|  | R insula |  | 4.9 | 39 | 5 | -11 |
|  | R putamen |  | 4.9 | 30 | -19 | 1 |
|  | R insula |  | 4.8 | 33 | -22 | 10 |
|  | R thalamus |  | 4.8 | 12 | -19 | 7 |
|  | R caudate |  | 4.8 | 21 | 26 | 1 |
|  | L rolandic operculum | 1008 | 5.7 | -57 | 2 | 13 |
|  | L rolandic operculum |  | 5.7 | -48 | -10 | 19 |
|  | L insula |  | 5.5 | -45 | 8 | 7 |
|  | L rolandic operculum |  | 5.5 | -51 | -7 | 13 |
|  | L insula |  | 5.2 | -27 | 26 | 13 |
|  | L putamen |  | 5.1 | -30 | -13 | -2 |
|  | L thalamus |  | 5.0 | -12 | -19 | 7 |
|  | L insula |  | 5.0 | -33 | -10 | 16 |
|  | L putamen |  | 4.9 | -18 | 14 | 4 |
|  | L putamen |  | 4.9 | -18 | 14 | 16 |
|  | L insula |  | 4.9 | -27 | 32 | 4 |
|  | L insula |  | 4.8 | -30 | 14 | 16 |
|  | L putamen |  | 4.8 | -21 | 8 | 10 |
|  | L putamen |  | 4.8 | -21 | 14 | 10 |
|  | L thalamus |  | 4.7 | -15 | -10 | 13 |
|  | L thalamus |  | 4.6 | -18 | -28 | 1 |
|  | L ant cingulate cortex | 7 | 5.0 | -15 | 44 | 13 |
|  | L med frontal gyrus (mid OFC) | 89 | 4.8 | -15 | 68 | -2 |
|  | L sup frontal gyrus (mid OFC) |  | 4.6 | -18 | 65 | -5 |
|  | L mid frontal gyrus (mid OFC) |  | 3.8 | -39 | 56 | -2 |
|  | L sup frontal gyrus (mid OFC) |  | 3.5 | -12 | 59 | -14 |
|  | L mid frontal gyrus (mid OFC) |  | 3.2 | -33 | 44 | -5 |
|  | R mid frontal gyrus (mid OFC) | 62 | 4.6 | 33 | 59 | -8 |
|  | R sup frontal gyrus (mid OFC) |  | 3.8 | 15 | 65 | -5 |
|  | R mid frontal gyrus (mid OFC) | | 3.5 | 45 | 47 | -14 |
|  | R inf frontal gyrus (lat OFC) |  | 3.4 | 54 | 44 | -11 |
|  | R mid frontal gyrus (mid OFC) | | 3.4 | 45 | 53 | -11 |
|  | R inf frontal gyrus (lat OFC) |  | 3.3 | 51 | 50 | -5 |
|  | R ant cingulate cortex | 71 | 4.6 | 15 | 44 | 19 |
|  | R ant cingulate cortex |  | 4.5 | 12 | 17 | 28 |
|  | R ant cingulate cortex |  | 4.3 | 18 | 44 | 7 |
|  | R ant cingulate cortex |  | 4.3 | 18 | 44 | 13 |
|  | R ant cingulate cortex |  | 4.3 | 15 | 50 | 13 |
|  | R ant cingulate cortex |  | 4.1 | 15 | 29 | 25 |
|  | R ant cingulate cortex |  | 3.9 | 12 | 47 | 22 |
|  | R ant cingulate cortex |  | 3.8 | 15 | 38 | 10 |
|  | R ant cingulate cortex |  | 3.7 | 12 | 29 | 16 |
|  | R ant cingulate cortex |  | 3.6 | 6 | 5 | 28 |
|  | L inf frontal gyrus (lat OFC) | 9 | 4.5 | -45 | 47 | -14 |
|  | L ant cingulate cortex | 13 | 4.1 | -9 | 14 | 28 |
|  | L caudate | 10 | 3.9 | -6 | 20 | -2 |
|  | L caudate |  | 3.9 | -12 | 23 | -5 |
|  | L caudate |  | 3.8 | -15 | 26 | -2 |
|  | L sup frontal gyrus (mid OFC) | 8 | 3.8 | -18 | 41 | -14 |
|  | R sup frontal gyrus (mid OFC) | 9 | 3.7 | 15 | 23 | -17 |
|  | R sup frontal gyrus (mid OFC) |  | 3.6 | 15 | 32 | -17 |
|  | R sup frontal gyrus (mid OFC) | 8 | 3.5 | 21 | 44 | -14 |
|  |  |  |  |  |  |  |

Activations were thresholded at p<0.001, with small volume correction over the ROI volume and a cluster extent threshold of k>4 contiguous voxels. Ant = anterior, sup = superior, inf = inferior, mid = middle, lat = lateral, med = median, L = left and R = right.
